# Shivering as a Diagnostic Sign of Thoracic Inflammation

**Published:** 1844-04

**Authors:** 


*Shivering as a diagnostic sign of Thoracic inflammation.*—M. CHOMEL, in one of his clinical lectures at the Hôtel Dieu, made the following remarks on this subject, in commenting on a case of pneumonia, that was in the wards:—

"I took much pains in questioning this patient, to ascertain whether she had experienced any chill before the commencement of the attack; and her reply was always in the negative. This circumstance appears to me of importance; and it is therefore designedly that I call your attention to the subject, seeing that it is the professed opinion of many physicians that pneumonia, like articular rheumatism, may generally be traced to the influence of damp and cold. The results, however, of my own experience, as well as of that of many others whom I know, are quite opposed to this opinion. No doubt it often happens, that pneumonic patients will be found to have been chilled some time before the attack came on; but assuredly, the chill is not the only, nor even the principal, cause of the disease. If we inquire into the particulars of a case, we generally find, that there was a predisposition to the malady present in the system at the time, and that the chill only accelerated the development of the mischief.

"It was merely the occasion, so to speak, of the explosion of a pre-existing morbid state; just in the same manner as a simple indigestion may be the exciting cause of a gastric inflammation in a person, in whom there is a strong disposition to this disease.

"But the same remark does not hold true of shivering when this occurs at the commencement of a disease. In my opinion, it is an almost invariable sign of pulmonary inflammation. Whenever, therefore, this symptom is or has been present, the physician will do wisely to direct his attention to the chest; and very generally, at least according to my experience, he will find that an inflammatory process has been set up in the lungs—unless indeed some well-marked symptoms clearly point to another organ as the seat of suffering. I do not deny, as a matter of course, that an attack of peritonitis, enteritis, &c., is sometimes ushered in with shivering; all that I mean to assert is, that this symptom is infinitely more common as a precursor of pneumonia than of any other inflammation. Hence in practice, whenever any of my patients has a well-marked shivering fit, even although other symptoms indicative of disease elsewhere be stronly marked, I at once suspect that the lungs are more or less seriously affected. On very many occasions, indeed, this symptom alone has sufficed to suggest to me the right diagnosis, while other medical men who have seen the case at the same time, have formed a very different opinion.

"There is another character which equally deserves the attentive consideration of the physician—and that is the pain in the side. In pleuro-pneumonia the pain is generally seated in the region of the mamma, although the affected part of the lung does not correspond to this point, or perhaps extends much beyond it. It has been suggested, in the way of explanation, that there is a greater degree of friction between the pulmonic and the costal pleuræ at this point than at any other, and that this may be the cause of the phenomenon in question. But if such were the case, the pain should surely not be limited to so circumscribed a spot, but should extend over all the surface where this greater friction is experienced; and we might expect, moreover, that it should change its locality—which certainly does not hold true. No satisfactory explanation has hitherto been offered of this symptom, and we must therefore confess our ignorance upon the point."—*Gazette des Hôpitaux.* *American Jour., Jan.* 1844.

